# Vapours of US and EU Market Leader Electronic Cigarette Brands and Liquids Are Cytotoxic for Human Vascular Endothelial Cells

**DOI:** 10.1371/journal.pone.0157337

**Published:** 2016-06-28

**Authors:** Raphaela Putzhammer, Christian Doppler, Thomas Jakschitz, Katharina Heinz, Juliane Förste, Katarina Danzl, Barbara Messner, David Bernhard

**Affiliations:** 1 Cardiac Surgery Research Laboratory, University Clinic for Cardiac Surgery, Medical University of Innsbruck, Innsbruck, Austria; 2 MCI Management Center Innsbruck, Biotechnology, Innsbruck, Austria; 3 Austrian Drug Screening Institute GmbH, Innsbruck, Austria; 4 Cardiac Surgery Research Laboratory, Department of Cardiac Surgery, Medical University of Vienna, Vienna, Austria; University of Sassari, ITALY

## Abstract

The present study was conducted to provide toxicological data on e-cigarette vapours of different e-cigarette brands and liquids from systems viewed as leaders in the e-cigarette market and to compare e-cigarette vapour toxicity to the toxicity of conventional strong high-nicotine cigarette smoke. Using an adapted version of a previously constructed cigarette smoke constituent sampling device, we collected the hydrophilic fraction of e-cigarette vapour and exposed human umbilical vein endothelial cells (HUVECs) to the mixture of compounds present in the vapour of 4 different single-use e-cigarettes, 6 different liquid vapours produced by the same refillable e-cigarette, and one e-cigarette with an exchangeable liquid cartridge. After incubation of cells with various concentrations and for various periods of time we analysed cell death induction, proliferation rates, the occurrence of intra-cellular reactive oxygen species, cell morphology, and we also measured e-cigarette heating coil temperatures. Overall, conventional cigarette smoke extract showed the most severe impact on endothelial cells. However, some e-cigarette vapour extracts showed high cytotoxicity, inhibition of cell proliferation, and alterations in cell morphology, which were comparable to conventional high-nicotine cigarettes. The vapours generated from different liquids using the same e-cigarette show substantial differences, pointing to the liquids as an important source for toxicity. E-cigarette vapour-mediated induction of oxidative stress was significant in one out of the 11 analysed vapours. There is a high variability in the acute cytotoxicity of e-cigarette vapours depending on the liquid and on the e-cigarettes used. Some products showed toxic effects close to a conventional high-nicotine cigarette. Liquid nicotine, menthol content, and the formation of acute intracellular reactive oxygen species do not seem to be the central elements in e-cigarette vapour toxicity.

## Introduction

The e-cigarette is generally promoted as “the healthier alternative” referring to a less harmful alternative to conventional cigarettes. The biggest market for e-cigarettes and liquids is the internet. By the end of 2014, 466 e-cigarette brands and 7764 types of e-liquid existed. [1–3] While international and national health legislation and regulation for e-cigarettes are still being developed, it has become clear that no reliable, objective and standardised systems are available yet that can test for e-cigarette liquid and vapour toxicity.

The e-cigarette generally consists of three components—a battery, a liquid cartridge (including the e-liquid), and the vaporizer i.e. a vaporization chamber including a heating element (also called “atomizer”)—and are sold as disposable or refill products with fixed or manually adjustable heating coil temperatures. Manufacturers`information guides often indicate a liquid vaporisation temperature between 40°C and 80°C. [1, 2, 4] However, the compounds which allow the generation of vapour have much higher boiling points: glycerol 290°C and propylene glycol 188.2°C. Currently, there are still no valid data available on temperatures applied to e-cigarette liquids, even though these temperatures are central to the formation of potentially hazardous compounds.

The second major element in e-cigarette vapour toxicity is the composition of liquids. Currently available liquid bases contain water, glycerol, propylene glycol, or mixtures of these compounds. Particularly, when heating the liquid base itself or the aroma compounds added within, new compounds may form, e.g. propylene oxide (from propylene glycol) or acrolein (from glycerol), which have well documented carcinogenic properties. [5–7] Many e-cigarette liquids contain nicotine and several liquids which are actually sold as “nicotine-free” liquids have, in fact, been found to contain nicotine. [8] Even though the toxicity associated with the nicotine concentrations delivered through most e-cigarettes may not be substantial, nicotine remains one of the most addictive substances known. The dosages of nicotine applied to the consumer when using e-cigarettes are unclear, not only because of ambiguous declaration of content by some producers/traders, but also because the vaporization process varies significantly between e-cigarette brands. Importantly, a standardised method for indicating nicotine concentration is lacking, resulting in incomparable information ranging from concentrations (mg/ml), total amount, to low-middle-high scales. [1, 4, 8, 9]

Similar to conventional cigarettes, e-cigarette liquids contain artificial flavouring. Some of these flavours are already known to be toxic. [7, 10] The effects of flavouring compounds, when modified by heating and/or interaction with other agents contained in the liquid is mainly unknown. The discovery of Tandalafil (virility promoting agent) and Rimonabant (appetite suppressant) in e-cigarette liquids [11] may indicate the urgent need for a better protection of consumers through quality control guidelines by national and international health institutions. [1, 2, 12, 13]

The third component in e-cigarette vapour toxicity is the vaporizer itself and materials used for constructing the hardware which come in physical contact with the user or the liquid. As an example, and probably as a central element in ‘hardware-based toxicity’, various metals have been shown to be released from the wire used as the heating element of the vaporizer. This wire is in permanent and direct contact with the e-liquid and, upon use, generates the vapour that is inhaled by the e-cigarette user. Metals such as aluminium, chromium, copper, lead, nickel, silver, and tin, have been found in e-cigarette vapour. [14, 15] and their relevance has previously been discussed in conventional cigarette smoke. [16, 17]

To date potential adverse effects of e-cigarette use on human health are not well defined as the products are still new and technology is changing rapidly. Particularly long term consequences of e-cigarette use are unclear.

This study was conducted to reveal potential acute cytotoxic effects of vapour generated by various, frequently sold, e-cigarette brands and liquids. Further the generation of reactive oxygen species was analysed and all effects were compared to the effects of a conventional cigarette. Finally we measured the temperatures that are produced by the heating coil of a leading brand vaporizer.

## Materials and Methods

### Materials

Based on an in-depth internet investigation, we selected e-cigarettes according to the following criteria: i) availability of a broad spectrum of different products (disposable e-cigarettes, e-cigarettes with a cartridge and e-cigarettes with refillable liquids), ii) products viewed as leaders in the European and USA e-cigarette market, iii) most popular flavours (tobacco, menthol, fruit flavours), and iv) liquids without as well as with various concentrations of nicotine. All liquids tested were vaporized with the identical vaporizer system. The high-nicotine conventional strength cigarette, as a reference, as well as the technology of smoke extract preparation has been reported previously. [18]

**NOTE:** the authors decided not to state e-cigarette producers, company names or product names, in order to avoid the misuse of our results on less harmful products for commercial purposes.

For a list of e-cigarette and liquid characteristics, see Table 1.

**Table 1:** Overview of e-cigarette brands analysed and characteristics of liquids.

System type	Brand/Type	Nicotine content	Liquid ingredients*
Disposable e-cigarettes	A/1	9 mg	Propylene glycol, glycerol, flavours, nicotine
	A/2	18 mg	Propylene glycol, glycerol, flavours, nicotine
	B/1	24 mg/ml	Propylene glycol, glycerol, flavours, nicotine
	C/1	yes: but not specified	
E-cigarettes for liquid refill	Cre1	6 mg/ml	Propylene glycol, glycerol, flavours
	Cre2	12 mg/ml	Propylene glycol, glycerol, flavours
	Cre3	6 mg/ml—menthol	Propylene glycol, glycerol, flavours
	Cre4	12 mg/ml—menthol	Propylene glycol, glycerol, flavours
	Cre5	0 mg/ml—cowberry	Propylene glycol, glycerol, flavours
	Cre6	0 mg/ml—absinthe	Propylene glycol, glycerol, flavours
e-cigarette with exchangeable cartridge	D	yes: but not specified	Propylene glycol, flavours, nicotine

*… indicated by the manufacturer/trader.

### Cigarette smoke and e-cigarette vapour extract generation

Cigarette smoke and e-cigarette vapour extracts were generated as previously described. [18] For e-cigarettes that required the vaporizer to be switched on manually, the operator of the smoke/vapour-generating machine pressed the “on button” on the e-cigarette 1 second prior to each of the automatic “drag phases” (which lasts for 2 seconds) of the machine and activated the e-cigarette until the end of the drag phase. For all cigarettes and liquids tested, the extracts were generated in the same manner; i.e. 20 cycles of 2 seconds dragging on the cigarette (35 ml / 2 seconds), followed by a 28 seconds pause. All e-cigarettes were fixed to a horizontal position while vapour was being generated. Importantly, all devices/tubes etc. in contact with smoke or vapour were exchanged between the extract preparation steps of each product and cleaned prior to re-use by intensive washing with ethanol (ultrapure) and water (ultrapure). The generation of vapour from identical amounts of liquid for all products tested was controlled by weighing the liquid cartridge (refill products) or the entire e-cigarette (single use devices) prior and after extract generation. Per generation of one extract (i.e. 20 puffs) a total of 88 +/- 5 mg of liquid was vaporized.

### Cell isolation and culture

The isolation and culture of HUVECs has been described previously. [19, 20] The isolation from umbilical veins and analysis of HUVECs used in this study was approved by the Ethics Committee of the Medical University of Vienna (No.: 1183/2012). Donors gave written informed consent on the use of tissue for cell isolation and culture. Cells were routinely passaged in 0.2% gelatine-coated (Sigma, Steinheim, Germany) polysterene culture flasks (TPP, Switzerland) in endothelial growth medium (EGM, Lonza) in a humidified atmosphere containing 5% CO_2_. Cells used for the experiments of this study were in passages 5 or 6.

### Exposure of cells to smoke and vapour extracts

Prior to exposure of cells to smoke or vapour extracts, cells were trypsinized, washed, counted and seeded into 6 well plates (300,000 cells per well for 1 and 24 hour exposures, and 150,000 cells per well for 48 hour exposures), and allowed to adhere overnight (for further details see [18, 21]). Prior to each experiment cell culture medium was replaced with fresh medium. For each experiment smoke and vapour extracts were freshly generated, filtered through a 0.2 μm filter, and applied directly to the cells by addition of the extracts to the culture plates to achieve the different % of extracts indicated. The extract concentrations chosen are based on our previous studies on conventional cigarettes [18, 21] with the assumption that e-cigarette consumers inhale a comparable volume of vapour to the volume of smoke inhaled by a conventional smoker (35 ml / puff, 10 puffs per cigarette). The system was previously adjusted to generate nicotine concentrations in the smoke extract which were similar to nicotine concentrations found in the blood of smokers. [18] As e-cigarette liquids do not contain a defined set of compounds which may allow for a standardisation, different extracts were compared by analysing the biological effects of equal amounts of the different vapour extracts. One hundred % extract corresponds to 20 times 35 ml of vapour drawn through 8 ml of culture medium.

### Quantification of cell death

For detection and quantification of cell death, forward/sideward light scattering analysis and annexin V (AxV) / propidium iodide (PI)-staining were used as described. [19, 20] AxV / PI-staining allows for the discrimination between intact viable cells (AxV-negative / PI-negative), apoptotic (AxV-positive / PI-negative) and necrotic cells (AxV-positive / PI-positive). After incubation of cells with e-cigarette extracts and cigarette smoke extracts for 24 hours (data not shown) and 48 hours, cells were stained and analysed using a FACS Calibur (BD, Vienna, Austria). The distribution of cells into the above groups was assessed; data are expressed as mean values and cells were categorized into either viable cells (AxV / PI-double negative cells) or dead cells (total cells minus AxV / PI double negative cells). The rationale for not differentiating between apoptotic and necrotic cells was that almost exclusively necrotic cell death was observed. [22] Results are expressed as % viable cells compared to the control.

### Analysis of cell proliferation

The proliferation of HUVECs was analysed using the CellTrace CFSE Proliferation Kit according to the manufacturer`s instructions (LifeTechnologies). Briefly, following staining of cells with the dye carboxyfluorescein diacetate succinimidyl ester (CFSE) and rinsing of non-bound dye, 1 x 10^5^ stained cells were seeded into each well of a 6 well plate. Following an overnight incubation and replacement of culture medium with fresh medium, cells were treated with the cigarette smoke- or e-cigarette vapour-extracts as indicated. After treatment periods of 24 hours (data not shown) and 48 hours, cells were washed, trypsinized, and subjected to CFSE quantification on a FACS Calibur by determination of mean fluorescence intensities of the viable cells. Mean fluorescence intensity of controls was set at 100% and the different incubations were calculated in relation to the corresponding control.

### Quantification of intracellular reactive oxygen species

The production and quantity of intracellular reactive oxygen species in HUVECs was assessed by measuring the oxidation of intracellular 2’, 7’-dichloro-dihydrofluorescein diacetate (H_2_-DCF-DA, Sigma-Aldrich, USA). The procedure using HUVECs has previously been described. [16] Briefly, 1 x 10^5^ cells per well of a 6 well plate were stained for 30 minutes with H_2_DCF-DA. Following washing of cells to remove excess dye, cells were incubated with the indicated concentrations of smoke/vapour extracts for 1 hour. Following trypsinisation of cells H_2_DCF-DA oxidation was quantified using a FACS Calibur.

### Analyses of cell morphology

To generate images of the morphological changes in endothelial cells exposed to e-cigarette vapour extracts, cells were cultured and seeded as described above. After allowing cells to adhere overnight and following 48 hour of incubation with the indicated concentrations of cigarette smoke / e-cigarette vapour extracts, images of the cells were taken using a Zeiss Axioscope 2 plus microscope (Zeiss, Germany) equipped with an Olympus AxioCam ERc 5s camera and an Axiovision Rel 48 software system.

### Measurement of the temperatures in a commercially available e-cigarette heating coil

For the analysis of temperatures which occur in a commercially available e-cigarette heating coil (not-adjustable), the vaporizer unit was opened and an infrared thermometer was applied (Mini Flash II, TFA Dostmann GmbH + Co. KG, Wertheim-Reicholzheim, Germany). Analyses were conducted on an e-cigarette with a fully charged battery in a ‘dry mode’, i.e. without liquid, and a ‘wet mode’, with liquid covering the entire heating coil. Temperatures were measured after various heating times (1, 2, 3, 4, 5, and 10 seconds). When the heating coil remains activated in the ‘dry mode’ the coil can be heated until a state of red-hot, which is at least several hundred to ~ 1000°C. Following each analysis the heating coil was cleaned using 2-propanol, and dried prior to each measurement. The liquid used for the ‘wet mode’ was 800μl of a e-liquid basis containing 60% propylene glycol, 30% glycerol, and 10% water (pH = 5) (1000ml VPG base zero, VAPE GmbH, Germany).

### Statistics

All data are shown as mean ± standard deviation (SD). Each experiment was performed at least 3 times with 3 parallels per experiment. Statistical analyses were conducted using IBM SPSS 20.0 software. All data were tested for Gaussian distribution and subsequently 1-way ANOVA was conducted using Sidak post hoc tests for multiple comparisons. A p-value <0.05 was determined as statistical significant (*…<0.05; **…<0.01; ***…<0.001).

## Results

### Five out of eleven e-cigarette vapours analysed show acute cytotoxicity

**Fig 1:**
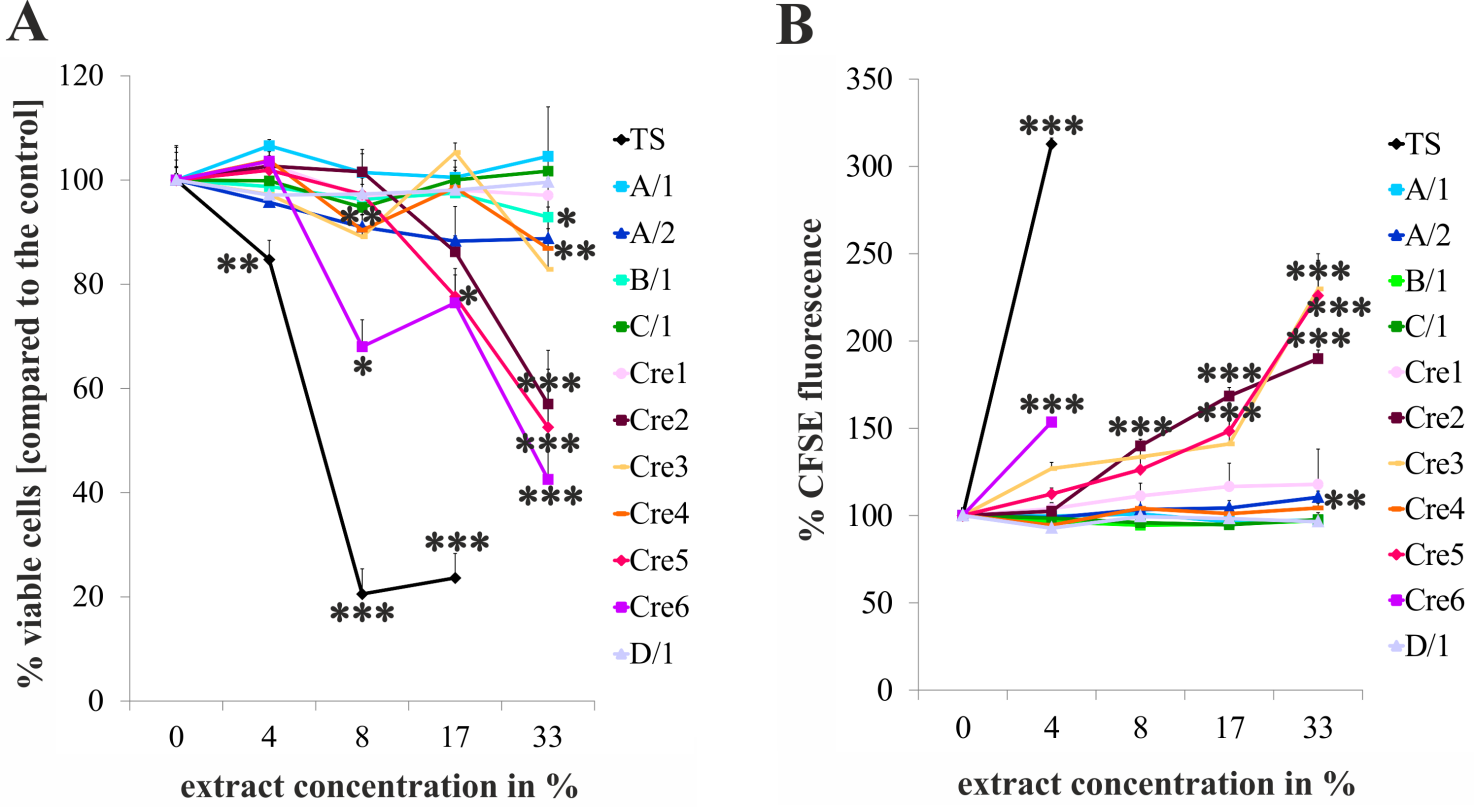
E-cigarette vapours are cytotoxic and inhibit cell proliferation of primary human endothelial cells. Fig 1 (A) shows the summary of FACS analyses of annexin V-propidium iodide stained HUVECs 48 hours after the addition of the indicated concentrations of conventional cigarette extract (TS) and different e-cigarette vapour extracts. Fig 1 (B) shows the effects of the same smoke extract and vapour extracts on the proliferation of endothelial cells after 48 hours of exposure analysed by CFSE staining and FACS analysis. A, B, C, and D reflect different market leader brands, and numbers label different products. Extracts labelled with “Cre” are vapour extracts from different liquids generated using the same liquid-refill vaporizer. A/1, A/2, B/1 and C/1 are single use e-cigarettes; D/1 is an e-cigarette with an exchangeable liquid cartridge. Data shown are mean values +/- S.D. of a representative performed in triplicates. The experiment was repeated three times. Asterisks indicate significant differences compared to the 0% (i.e. the control) p<0.05.

In order to reveal potential cytotoxic effects of e-cigarette vapours from various liquids, we generated e-cigarette vapour extracts and exposed HUVECs to these vapours for 48 hours. These analyses showed that 5 (B/1, Cre2, Cre4, Cre5, Cre6) out of 11 e-cigarettes or e-liquids tested showed a statistically significant increase in cell death (see Fig 1A), two of them already at a lower concentration of 8% (Cre4 and Cre6). Another major finding of these analyses was that 3 (Cre2, Cre 5, Cre6) out of 6 liquids which were vaporized using the same system showed dramatic cytotoxic effects, and reached toxicity close to conventional strength high-nicotine cigarettes. Of interest is also that 2 (Cre5 and Cre6) of these 3 highly toxic liquids did not contain nicotine, but were flavoured with berry or herbal constituents. In this study also data for 24 hours were obtained. As these 24 hour data were similar to the results after 48 hours of incubation they are not shown. For detailed results of statistical analyses also see Table A in S1 File.

### Five out of eleven e-cigarette vapours reduce the proliferation of endothelial cells

The analyses of the proliferation inhibiting activity of e-cigarette vapour extracts revealed that 5 (A/2, Cre2, Cre3, Cre5, Cre6) out of 11 e-cigarettes or e-liquids tested showed a statistically significant reduction of cell proliferation (see Fig 1B). Importantly, toxicity of chemicals is a major reason for reduced proliferation of cells, and the reduction of proliferation–in case of toxicity–is a more sensitive parameter compared to cell death, as it already occurs at lower concentrations of those chemicals. In case of the 11 e-cigarette vapours analysed, the indication of toxicity as the source for reduced proliferation can well be observed as the three extracts with the highest cytotoxicity (Cre2, Cre5, and Cre6, see above) are also the extracts which most potently inhibited cell proliferation. Interestingly, although no significant increase in cell death extent was observable after incubation with Cre3 e-liquid, the proliferation of HUVECs is significantly reduced by this liquid. In this study also data for 24 hours were obtained. As these 24 hour data were similar to the results after 48 hours of incubation they are not shown. For detailed results of statistical analyses also see Table B in S1 File.

### One out of 11 e-cigarette vapours cause an increase in intracellular reactive oxygen species

**Fig 2:**
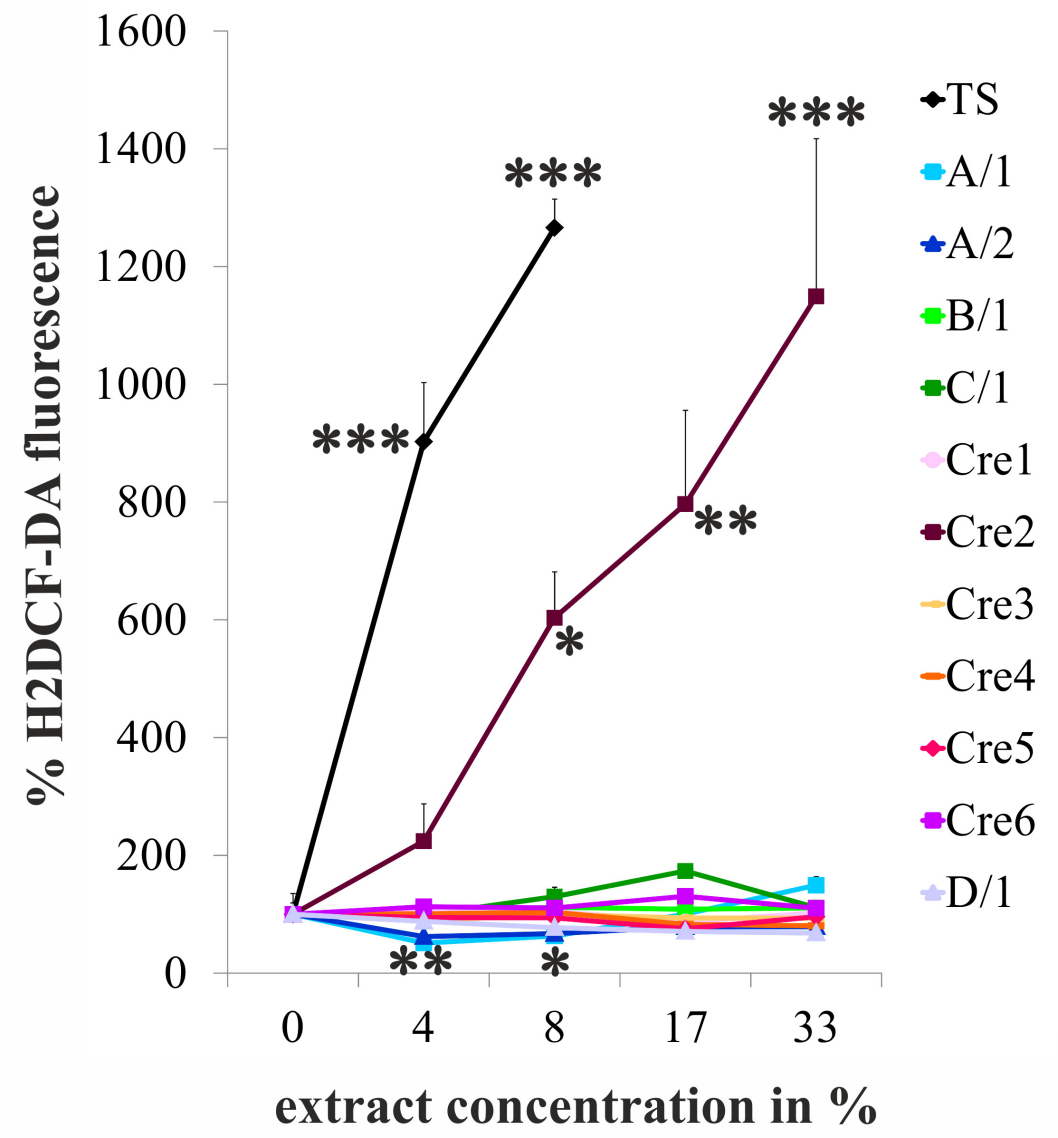
The induction of intracellular reactive oxygen species does not correlate with e-cigarette vapour toxicity. Fig 2 shows an analysis of the formation of intracellular reactive oxygen species in HUVECs 1 hour after exposure to cigarette smoke extract (TS) and e-cigarette vapour extracts and concentrations indicated. MFI…mean fluorescence intensity of the oxidation-sensitive dye H2DCF-DA. A, C, and D reflect different market leader brands, and numbers label different products. Data shown are mean values +/- S.D. of a representative experiment performed in triplicates. The experiment was repeated three times. Asterisks indicate significant differences compared to the control (0%) p<0.05.

Oxidative stress is a potent mechanism for cytotoxicity and for the inhibition of proliferation, thus being a central element in the pathogenic activity of conventional cigarettes. In the present study only 1 out of the 11 e-cigarette vapour extracts tested was found to cause the formation of reactive oxygen species in HUVECs, one hour after exposure (see Fig 2). Generally, the occurrence of early/immediate reactive oxygen species was not correlated to the induction of cell death or impaired proliferation. Only Cre2, which is among the most toxic extracts, induced reactive oxygen species formation. In contrast, e-liquid A/2 significantly reduced H2DCF-DC fluorescence compared to control at low extract concentrations. For detailed results of statistical analyses also see Table C in S1 File.

### Toxic e-cigarette vapour extracts cause morphological alterations in endothelial cells similar to conventional cigarette smoke extracts

**Fig 3:**
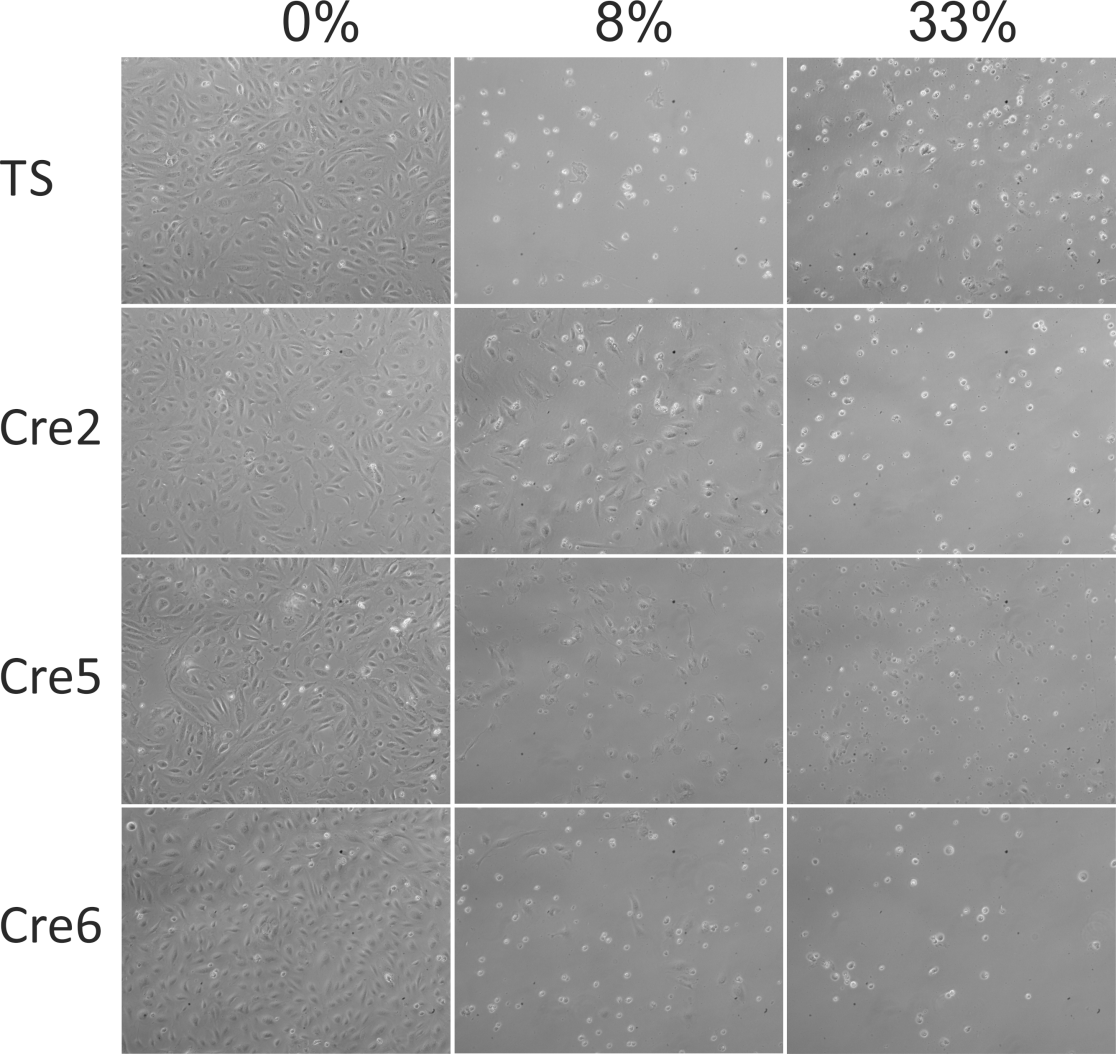
Vapour extracts alter the morphology of endothelial cells. The images in Fig 3 show HUVECs after 48 hours of exposure to the indicated extracts and concentrations. TS…conventional cigarette smoke extract. Cre2, 5 and 6 are the most cytotoxic liquid vapours according to the results shown in Fig 1. The experiment was repeated three times. Representative images are shown.

In a previous study we could show that conventional cigarette smoke extracts disrupt the vascular endothelial barrier function by breaking up cell-cell contacts and by inducting a collapse of the microtubule system, both leading to a detachment like phenotype [16]. In order to analyse the impact of e-cigarette vapour extracts on endothelial shape, cells were treated with vapour extracts of Cre2, Cre5, and Cre6. As can be seen in Fig 3, similar to conventional cigarette smoke extracts, also e-cigarette vapours cause significant morphological alterations in endothelial cells and disrupt the functional endothelial monolayer, represented by the controls (0%). Whereas high concentrations of vapour extracts are toxic to endothelial cells (see Fig 1), which leads to death induced detachment of cells, non- or slightly toxic concentrations (8%) also cause morphological changes which will certainly contribute to disease-induction by e-cigarette vapours.

### Temperatures of a commercially available non-adjustable e-cigarette heating coil

In order to obtain information on the “true” temperatures that are used in e-cigarettes we opened the vaporizer of a supplier viewed as leaders in e-cigarette market and analysed temperatures with an infrared thermometer under “dry” i.e. without liquid, and “wet” i.e. with liquid conditions. Under dry conditions the maximum temperature that can be analysed by the thermometer used (i.e. 217°C) was reached after 8.9 +/- 0.2 seconds. When the coil remained in the ‘on’ mode, the coil reached a red-hot state (i.e. at least several hundred °C). In the wet mode after 10 seconds of heating the maximum temperature reached was 116 +/- 3.9°C. Importantly, the vapour that is generated around the heating coil during the heating process may significantly affect measurements (i.e. the true temperatures may be higher than the measured temperatures). Due to the fact that the vaporizer had to be opened for temperature measurements, a drawing on the e-cigarette (which causes airflow) was not possible.

### The colour of some e-liquids changes over time

**Fig 4:**
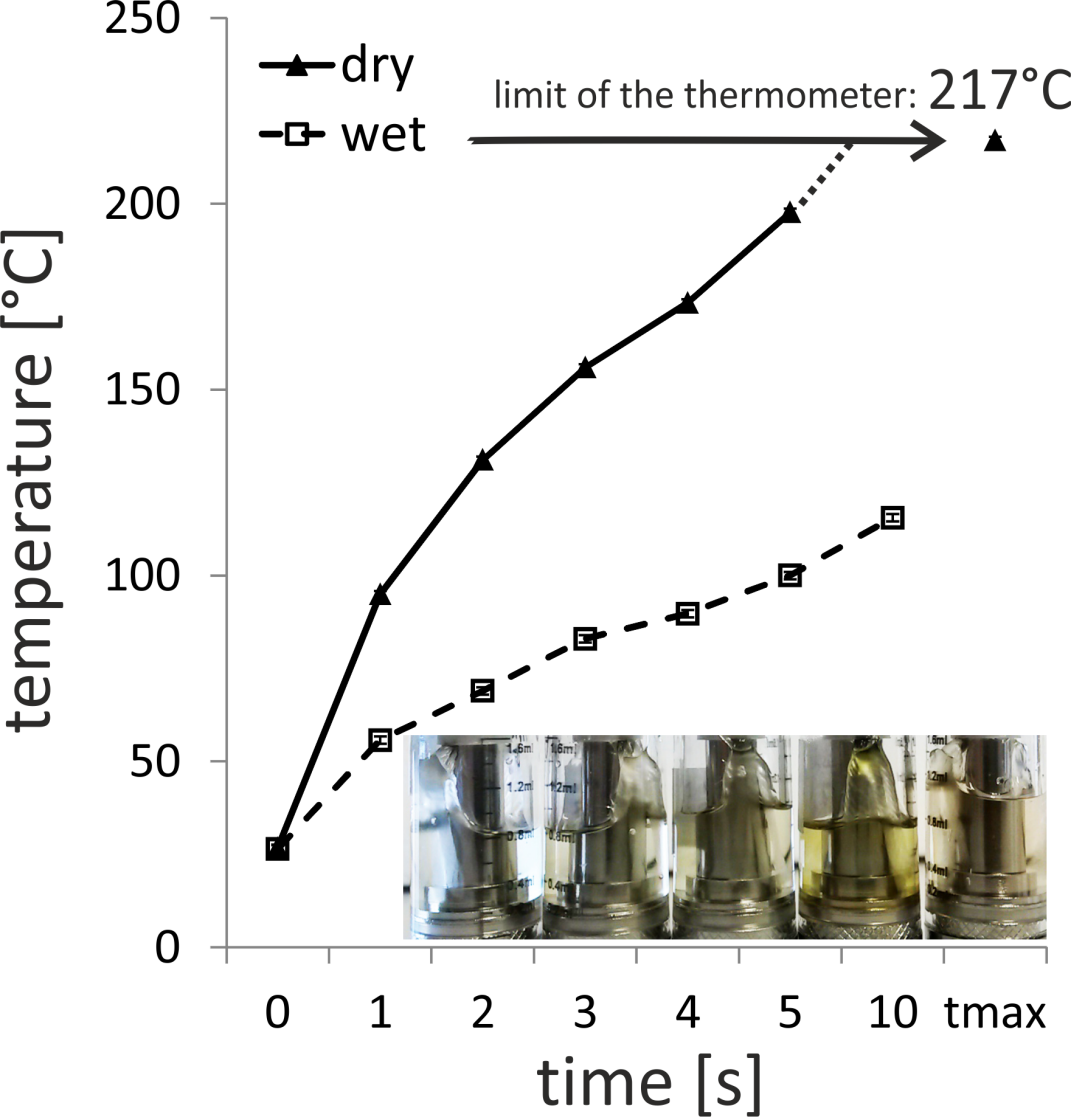
Assessment of the temperature of the heat coil of a commercially available vaporizer. The diagram in Fig 4 shows the two temperature curves of the heating element in ‘dry mode’ and ‘wet mode’; the broken line reflect the temperatures of the coil when surrounded by liquid (wet), the continuous line reflects the temperatures measured in the absence of a liquid (dry). The experiment was repeated three times. Data shown are mean values +/- S.D. Temperature was analysed using an infrared thermometer (max temperature (tmax) that can be measured using the device is 217°C). The image in the lower right of the diagram shows a photograph of different liquids, which remained in the liquid container during several times of e-cigarette vapour extract generation. All liquids were initially clear and colourless. Examples are shown.

As the liquid constituents are in permanent contact to e-cigarette components including the heating coil and are exposed to high temperatures and oxygen, chemical alterations of compounds may occur. As can be seen in the image in Fig 4, taken from different liquids used in this study to generate vapour extracts, some, but not all, liquids change their characteristics from initially: clear and transparent, to a yellow, brownish colour and increased turbidity.

## Discussion

### The effects of e-cigarette vapour on cells and tissues

An increasing number of valid and excellent scientific publications are available on the role and dangers of e-cigarette use, as a potential tool for cessation, and also in the field of toxicity. Until now there are important, yet few, studies available which analysed e-cigarette vapour toxicity, the majority of which support the view that e-cigarette vapour is toxic. [23–26] Previous studies have revealed e-cigarette-mediated alterations in glucose and lipid metabolism, also by liquids free of nicotine [27], an impact on the transcriptome of bronchial epithelial cells involving alterations in glycerophospholipid metabolism [28], cytotoxicity and pro-inflammatory signalling due to flavour-derived reactive oxygen species inhalation in the lung [25], and cell death and pro-inflammatory cytokine release by lung and skin derived cell lines [23]. Oxidative stress and inflammation are processes which are also thought to be central for the cytotoxicity of conventional cigarette smoke. [29] E-cigarette vapour specific processes and cytotoxicity are not well understood. Clearly this lack of information is due to the fact that e-cigarette toxicity research is a young field of research, but is also influenced by the fact that different liquids contain different toxic compounds with differing cytotoxic profiles–a view that is supported by studies by Lerner et al. and Behar et al. [25, 30] and the present study. It was the goal of the present study to obtain data on the cytotoxic and proliferation-inhibiting effects of e-cigarette vapours from different brands and liquids–all of which are currently considered as market leader products. By analysing cell death, proliferation, the formation of reactive oxygen species, by morphological analyses of HUVECs, and by choosing different sets of e-cigarettes and liquids (e.g. with and without nicotine, menthol etc.), this study allows new insights on potential pathogenic mechanisms and the role of some ingredients–certainly with limitations. Given the fact that there are currently almost 10,000 different liquids available [1, 2], this study (analysing 11 different vapours) is limited in its validity. Nevertheless–due to the choice of market leader products–basic results are of relevance.

### Central findings of the present study

1. The highest toxicity of all liquid extracts tested is found in liquid-refill devices. Of great importance is the observation that toxicity and inhibition of proliferation was highly dependent on the type of liquid used. For comparison see results for Cre1 to 6 (Figs 1 and 2).
2. Disposable products seem to be less toxic. Importantly, the “satisfactory feeling” of the user, when consuming e-cigarette vapour, was not assessed in this study. When adapting results from conventional cigarettes and conventional smokers, where it was shown that smoking behaviour is changed unconsciously due to the use of “light cigarettes” [31], similar subconscious traits may occur in e-cigarette use. The analysis of toxicity and compounds in e-cigarette vapour under different conditions of e-cigarette use shall be a central part of future studies.
3. Despite the fact that nicotine is the central reason for tobacco/cigarette (e-cigarette) dependence, vapour-extract toxicity does not correlate with nicotine content. Other studies suggest however, that nicotine may alter cellular responses and activate additional, potentially pathologically relevant signalling pathways. [27, 28] In the present study no correlation between the menthol content and toxicity was observed. Importantly, these statements are based on information provided by the manufacturers, and since the majority of liquid ingredients are not disclosed by companies, and not known by the authors, these statements need to be verified by chemical analyses of liquids by unbiased scientists.
4. In our study, particularly herbal flavours seem to contain highly toxic compounds; Plant extracts contain a huge number of different compounds, the nature of the toxicity-relevant compounds is unknown. Previously, similar effects were observed with cinnamon flavoured liquids. [30]
5. The formation of intracellular reactive oxygen species by e-cigarette vapour extracts was not correlated to their toxicity in our study. Lerner et al. showed however, that reactive oxygen species and copper (which catalyses the formation of reactive oxygen species) may contribute to e-cigarette smoke-mediated inflammation in the lung. [15, 25] Despite the fact that the role of reactive oxygen species is not clear, cell type specific processes may be at play. Based on our results, one would speculate that toxicity mechanisms of some e-cigarette vapours in endothelial cells may differ from those of conventional cigarettes, where reactive oxygen species are central for disease induction. [32]
6. The analysis of vaporizer heating coil temperatures during use, and the optical characteristics of e-liquid which remains in the cartridge, suggest that temperatures that are applied to liquids in e-cigarettes may be significantly higher than proposed by manufacturers (also see [33]), and that some liquids upon use undergo chemical modifications (aging/oxidation processes, similar to the Maillard reaction), and tend to precipitation or may even facilitate microbial growth.

### Conclusions

Given the fact that at least one out of 11 products tested herein was close to the toxicity of a conventional strength high-nicotine cigarette in the absence of high levels of reactive oxygen species, e-cigarette vapour-mediated patho-mechanisms and e-liquid (vapour) contained compounds are likely to impact human health in a way that differs from conventional cigarettes. This hypothesis is of particular interest regarding long term health effects of e-cigarette use.

Generally, e-cigarette vapour constituents affect cell viability, proliferation, morphology, metabolism, and state of inflammatory activation. [23, 25–28] All these alterations show that chemicals found in e-cigarette vapour influence cells in a fundamental way and broad manner. Based on the current knowledge, a precise definition of diseases that may be caused by chronic exposure to e-cigarette vapours in humans is not possible, but current data allow stating that e-cigarette use is indeed harmful.

### Limits of the study

Finally, it is important to the authors of this project to summarize the limitations of the study.

First, and clearly, facing the enormous number of available e-cigarettes and liquids the number of e-cigarettes and liquids analysed in this project is small, causing the need for confirmation of all conclusions and statements in future studies.

Second, this study did not test for potentially mutagenic effects of e-cigarette vapours and did not test for possible microbiological contamination of liquids and vapours. Further, this study cannot provide data on long term adverse health effects of e-cigarette vapour, and its acute toxicity tests are limited to power of the assays and cell type used.

Third, the conclusions drawn in this study partly rely on e-cigarette and liquid manufacturer’s information e.g. on nicotine or menthol content of liquids etc., and are not confirmed by own or other`s unbiased research.

Fourth, all tests, including vapour extract generation were performed under standardised conditions, which may lead to a bias as e-cigarettes or different liquids may be consumed in different manners by users. In line, extracts were generated by placing all e-cigarettes in a horizontal position–the position how the e-cigarette is held by the user is known to significantly affect the temperatures (wet vs. dry) and consequently the chemicals that are generated from the liquid constituents.

Fifth and finally, the conventional cigarette that was used as a toxicity-reference is a common strength high-nicotine cigarette. Accordingly, it seems likely that the comparison between conventional cigarette versus e-cigarette toxicity, when compared to other less strong conventional cigarettes (‘light cigarettes’), would result in the conclusion that e-cigarettes and vapours are more toxic than suggested by the results of this study.

## Supporting Information

S1 File This is the online supplement to this publication. (DOCX)

S2 File This are the raw data to the data and figures presented in this publication. (XLSX)
