# Supplementary material for: Vapours of US and EU Market Leader Electronic Cigarette Brands and Liquids Are Cytotoxic for Human Vascular Endothelial Cells
Source: PLoS One. 2016 Jun 28;11(6):e0157337. doi: 10.1371/journal.pone.0157337 (PMC4924852; doi:10.1371/journal.pone.0157337)
Supplement: S1 File — (DOCX) [file pone.0157337.s001.docx]

**S1 - ONLINE SUPPLEMENT**

**to**

**Vapours of US and EU Market Leader Electronic Cigarette Brands and Liquids are Cytotoxic for Human Vascular Endothelial Cells**

**by Putzhammer R. et al**

Correspondence to:

David Bernhard, Ass.-Prof. Priv.-Doz. Mag. Dr.

Cardiac Surgery Research Laboratory

Medical University of Innsbruck

Innrain 66A, 5th floor, room 15

A-6020 Innsbruck, AUSTRIA

Phone: 0043-(0)512-504-26291

Fax: 0043-(0)512-504-26292

E-mail: david.bernhard@i-med.ac.at

**Material and Methods**

All data are shown as mean ± standard deviation. Each experiment was performed at least 3 times with 3 parallels per experiment. Statistical analyses were conducted using IBM SPSS 20.0 software. All data were tested for Gaussian distribution and for mean comparisons between multiple groups subsequently 1-way ANOVA was conducted using Sidak post hoc tests for multiple comparisons. A p-value <0.05 was determined as statistical significant (*...<0.05; **…<0.01; ***…<0.001).

**Results**

To analyse statistical significant differences, ANOVA with Sidak post hoc test for multiple comparisons was performed and the calculated p-values are indicted in the Tables A, B and C of the S1 Online Supplement. In detail Table A – S1 Online Supplement shows statistical evaluations for extract induced cell death compared to the corresponding control. Statistical analyses were also performed for proliferation quantifications in control and extract incubated HUVECs and the p-values are depicted in Table B – S1 Online Supplement. Likewise the extent of extract induced oxidative stress in terms of increased H2DCF-DC fluorescence was statistically analysed and the p-values compared to the corresponding controls are shown in Table C – S1 Online Supplement.

**Table A - S1 Online Supplement: Statistical analyses and indication of p-values for Annexin V/PI staining results using ANOVA test (Sidak post hoc test).**

|  | **concentration** | | | | |
| --- | --- | --- | --- | --- | --- |
| **extract** | **0%** | **4%** | **8%** | **17%** | **33%** |
| **TS** |  | 0.004 | <0.001 | <0.001 | nd |
| **A/1** |  | ns | ns | ns | ns |
| **A/2** |  | ns | ns | ns | ns |
| **B/1** |  | ns | ns | ns | 0.022 |
| **C/1** |  | ns | ns | ns | ns |
| **Cre1** |  | ns | ns | ns | ns |
| **Cre2** |  | ns | ns | ns | <0.001 |
| **Cre3** |  | ns | ns | ns | ns |
| **Cre4** |  | ns | 0.035 | ns | 0.004 |
| **Cre5** |  | ns | ns | 0.041 | <0.001 |
| **Cre6** |  | ns | 0.016 | ns | <0.001 |
| **D/1** |  | ns | ns | ns | ns |

ns = not significant; nd = not determined

**Table B - S1 Online Supplement: Statistical analyses and indication of p-values for CFSE staining results using ANOVA test (Sidak post hoc test).**

|  | **concentration** | | | | |
| --- | --- | --- | --- | --- | --- |
| **extract** | **0%** | **4%** | **8%** | **17%** | **33%** |
| **TS** |  | <0.001 | nd | nd | nd |
| **A/1** |  | ns | ns | ns | ns |
| **A/2** |  | ns | ns | ns | 0.009 |
| **B/1** |  | ns | ns | ns | ns |
| **C/1** |  | ns | ns | ns | ns |
| **Cre1** |  | ns | ns | ns | ns |
| **Cre2** |  | ns | <0.001 | <0.001 | <0.001 |
| **Cre3** |  | ns | ns | ns | <0.001 |
| **Cre4** |  | ns | ns | ns | ns |
| **Cre5** |  | ns | ns | 0.001 | <0.001 |
| **Cre6** |  | <0.001 | nd | nd | nd |
| **D/1** |  | ns | ns | ns | ns |

ns = not significant; nd = not determined

**Table C - S1 Online Supplement: Statistical analyses and indication of p-values for H2DCF-DC staining results using ANOVA test (Sidak post hoc test).**

|  | **concentration** | | | | |
| --- | --- | --- | --- | --- | --- |
| **extract** | **0%** | **4%** | **8%** | **17%** | **33%** |
| **TS** |  | <0.001 | <0.001 | nd | nd |
| **A/1** |  | ns | ns | ns | ns |
| **A/2** |  | 0.009↓ | 0.024↓ | ns | ns |
| **B/1** |  | ns | ns | ns | ns |
| **C/1** |  | ns | ns | ns | ns |
| **Cre1** |  | ns | ns | ns | ns |
| **Cre2** |  | ns | 0.018 | 0.002 | <0.001 |
| **Cre3** |  | ns | ns | ns | ns |
| **Cre4** |  | ns | ns | ns | ns |
| **Cre5** |  | ns | ns | ns | ns |
| **Cre6** |  | ns | ns | ns | ns |
| **D/1** |  | ns | ns | ns | ns |

ns = not significant; nd = not determined; ↓ = significant reduction of H2DCF-DC fluorescence
